# Supplementary material for: Healthcare-seeking behaviors and factors influencing non-adherence among cervical cancer patients attending Bugando Oncology Clinic in Mwanza, Tanzania: A qualitative Phenomenological study
Source: PLoS One. 2025 Mar 26;20(3):e0317609. doi: 10.1371/journal.pone.0317609 (PMC11940420; doi:10.1371/journal.pone.0317609)
Supplement: S1 File — (PDF) [file pone.0317609.s001.pdf]

Appendix I (b): Consent Form - English Version

CATHOLIC UNIVERSITY OF HEALTH AND ALLIED SCIENCES-BUGANDO
DIRECTORATE OF RESEARCH AND PUBLICATIONS

Consent form for cervical cancer patients attending Bugando Oncology Clinic in Mwanza, Tanzania, and whom we are inviting to participate in research titled "Exploring healthcare-seeking behaviors and factors influencing non-adherence among cervical cancer patients attending Bugando Oncology Clinic in Mwanza, Tanzania."

Investigator: Mr. Bashari. N. Kidaya

Institution: The Catholic University of Health and Allied Sciences (CUHAS) - Bugando.

This Informed Consent Form has two parts:

- **Information Sheet (to share information about the study with you)**
- **Certificate of Consent (for signatures if you choose to participate)**

You will be given a copy of the full Informed Consent Form

Part I: Information Sheet

Introduction

I am Bashari. N. Kidaya, currently pursuing a Master's degree in Public Health (MPH) at the Catholic University of Health and Allied Sciences. I am researching cervical cancer, a prevalent disease in our country and region. I would like to provide you with information about the research and extend an invitation for you to participate. Please note that you are not obligated to make a decision today regarding your participation. You are encouraged to discuss the research with

someone you trust before making a decision. If you come across any unfamiliar terms or concepts in this consent form, please feel free to ask me to pause and I will take the time to explain. Should you have any questions later on, you can direct them to me or another researcher involved in the study.

Purpose of the research: Cervical cancer has been affecting numerous women in our community, and we are determined to find ways to prevent its occurrence. We believe that your contribution can greatly assist us in understanding your knowledge about cervical cancer and your general healthcare-seeking behaviors. We aim to explore the healthcare-seeking behaviors and factors affecting non-adherence among cervical cancer patients attending Bugando Oncology Clinic. Additionally, we seek to learn about the preventive measures women employ to reduce the incidence of cervical cancer and how the community recognizes its presence. Furthermore, understanding healthcare-seeking behaviors and factors influencing non-adherence will enable us to develop effective strategies to control the disease in our community.

Type of Research Intervention: This research will require your participation in an in-depth interview, which is expected to last approximately 30 to 45 minutes.

Participant Selection: You are being invited to participate in this research because your experience as a cervical cancer survivor is highly valued and can greatly contribute to our understanding of healthcare-seeking behaviors and factors influencing non-adherence.

Voluntary Participation: Your participation in this research is completely voluntary, and the decision to participate or not is entirely up to you. If you choose not to participate, please be assured that all the services you receive at this Centre will continue without any changes.

Procedures: We kindly request your assistance in helping us gain a deeper understanding of cervical cancer within your community. Should you choose to accept, you will be invited to engage in an interview conducted by a research assistant. The interview will take place at your household or any comfortable setting of your suggestion. During the interview, if at any point you feel uncomfortable answering a question, you may simply express your preference, and the interviewer will move on to the next question. Unless you desire otherwise, only the interviewer will be present during the interview. Please be aware that all information shared during the interview will be treated with utmost confidentiality, and the research team will have access to the information documented. The entire interview will be audio-recorded; however, no individual will be identified by name on the recording. The recordings will be stored securely in a locked cabinet. Rest assured that the information shared is strictly confidential and will not be accessible to any individuals other than the research team. After two months, once the data is no longer needed, the recordings will be destroyed.

Duration: The research will be conducted over a total period of two months. Within this timeframe, we will arrange a single visit to conduct an interview with you, which is expected to last approximately 30 to 45 minutes.

Risks: We understand that the information we are requesting may be personal and confidential, and discussing certain topics might make you uncomfortable. You need to know that you have the right to choose not to answer any questions or participate in the interview if you do not wish to do so. Your decision will be respected, and you are not obligated to provide any explanation for not responding to specific questions or declining to participate in the interview.

Benefits: While there may be no direct personal benefit to you, your participation in this research is highly valuable as it can contribute to our understanding of preventing and treating cervical cancer within your community.

Reimbursements: You will not receive any incentives for participating in the research. However, as a token of appreciation for your time, we would like to offer you a 1-liter bottle of water.

Confidentiality: The research conducted within the community setting may attract attention, and as a participant, you may be approached by other individuals in the community with questions. Please be assured that we will not share any information about you with anyone outside of the research team. The data collected during this research project will be treated as confidential. Your personal information will be identified by a unique number instead of your name. Only the researchers will have access to the key that links your number to your identity, and this information will be securely stored. Under exceptional circumstances, such as a request from the Research Ethics Committees, the information may be shared, but only by strict confidentiality protocols.

Sharing the Results: Any information you provide today will remain strictly confidential and will not be shared with anyone outside the research team. Your identity will not be disclosed in any reports or publications. The knowledge gained from this research will be shared with you, as well as with the appropriate university and hospital authorities before it is disseminated to the public. Each participant will receive a summary of the research findings. Meetings will be organized at the university and hospital where the results will be published to allow others who are interested to learn from the research. These events will be announced to ensure transparency.

Right to Refuse or Withdraw: Participation in this research is entirely voluntary, and you have the right to decline or withdraw from the study at any time without any impact on the services you

receive at Bugando Medical Centre. You will have the opportunity to review your interview responses at the end, and if you disagree with any of the recorded information or feel that I may have misunderstood you, you can request modifications or removal of those sections.

Who to Contact: If you have any questions, please feel free to ask them now or at any time during the research process. Alternatively, you can contact the principal researcher of this study, Mr. Bashari. N. Kidaya, at the Catholic University of Health and Allied Sciences, P.O. Box 1464 Mwanza, Tanzania. You can also reach him via mobile phone at 0766298594 or through email at chriskidaya@gmail.com.

This proposal has undergone a thorough review and has been approved by both the CUHAS and BMC Research and Ethics Review Committee. This committee is responsible for ensuring the protection of research participants from any potential harm. If you would like to learn more about the committee, you may contact the Chairman of the CUHAS and BMC Research and Ethics Review Committee through the following address: P.O. Box 1464, Mwanza, Tanzania, or via telephone at 28-298 3386.

Part II: Certificate of Consent

I have been invited to participate in research about healthcare-seeking behaviors, and factors influencing non-adherence among cervical cancer patients. I have read the foregoing information, or it has been read to me. I have had the opportunity to ask questions about it and any questions I have been asked have been answered to my satisfaction. I consent voluntarily to be a participant in this study.

Name of Participant ____

Signature of Participant ____________________ **Date** ____________ (day/month/year)

If illiterate I have witnessed the accurate reading of the consent form to the potential participant, and the individual has had the opportunity to ask questions. I confirm that the individual has given consent freely.

Name of witness __________________________ Thumbprint of participant

Signature of witness _______________________ Date __________________ (day/month/year)

(A witness should be selected by the participant)

Statement by the researcher/person taking consent

I have accurately read out the information sheet to the potential participant, and to the best of my ability made sure that the participant understands that their responses will be recorded, either in audio or written form, and that these records will be used for analysis and reporting.

I confirm that the participant was allowed to ask questions about the study, and all the questions asked by the participant have been answered correctly and to the best of my ability. I confirm that the individual has not been coerced into giving consent, and the consent has been given freely and voluntarily.

A copy of this ICF has been provided to the participant.

Name of person taking the consent ____

Signature of person taking the consent _________________ Date ________ (day/month/year)
