# Supplementary material for: Healthcare-seeking behaviors and factors influencing non-adherence among cervical cancer patients attending Bugando Oncology Clinic in Mwanza, Tanzania: A qualitative Phenomenological study
Source: PLoS One. 2025 Mar 26;20(3):e0317609. doi: 10.1371/journal.pone.0317609 (PMC11940420; doi:10.1371/journal.pone.0317609)
Supplement: S2 File — (PDF) [file pone.0317609.s002.pdf]

Appendix ii (a): Interview Guide - English Version

"For non-adherent cervical cancer patients attending Bugando Oncology Clinic"

Identification number – BMC Onco 01 Date for an in-depth interview – 29/08/2023

A. Participant's Socio-demographic Information

1. Age (years) – 51
2. Highest education level reached – Standard 03
3. Occupation – Small business in the market
4. Religious denomination – Roman Catholic
5. Marital status – Widow
6. Residence (district, region) – Sirari, Mara

B. Exploring of healthcare-seeking behaviors

1. Nowadays there are many health problems affecting people of the lake zone, and cancer is on the increase, mention types of cancers?

   R: Cervical cancer, Breast cancer

Probe: (If cervical cancer is not mentioned) How about cervical cancer?

2. What are the symptoms of cervical cancer?

   R: Stomach pain, heavy bleeding, sensing a tumor in my reproductive organs

Probe: What symptoms or concerns led you to seek healthcare for your cervical cancer?

   R: Stomach pain and heavy bleeding

3. Tell me any instances where you tried to treat yourself for cervical cancer or its symptoms.

   R: I never tried to treat myself, I went straight to the hospital in Kenya, and they injected me a syringe to stop bleeding and gave me some drugs, the bleeding stopped. After a year I felt

stomach pain and heavy bleeding, I went back to the same hospital and they told me it is cancer, and then they referred me to a hospital in Musoma.

Probe: What led you to believe that self-treatment was necessary or preferable?

4. What other alternative or complementary treatments have you sought?

   R: I did not tried any alternative treatments

Probe: Please, explain your experiences with these treatments.

Probe: How did you make a decision about which treatment to pursue?

5. What barriers or challenges have you faced in seeking healthcare for your cervical cancer?

   R: Moving to and from a hospital in Musoma several times to seek diagnosis, and waiting for six weeks have my final diagnosis, there is when the referred me to Bugando Medical Centre.

Probe: How have you managed to overcome those challenges?

   R: I just wait until I got my diagnosis

6. Describe sociocultural factors that have influenced your decision-making for cancer care.

   R: My children told me, mum let's take you to the hospital, as I don't have a man, it is just me and my children.

Probe: What specific misconceptions you have heard about cervical cancer treatment?

   R:

7. What advice would you give to other women who may be experiencing symptoms or concerns related to cervical cancer?

   R: I will advise them to seek healthcare at the hospital

8. Is there anything else you would like to share about your experience with seeking healthcare for your cervical cancer?

   R: Nothing

C. Identifying factors influencing non-adherence

1. What were your initial thoughts and feelings when you were diagnosed with cervical cancer?

   R: I felt too much pain, I saw like my life has ended. You know, in my region this disease is not familiar, everyone I shared the information with that I have this disease, they felt afraid of me. This made me felt much nervous and thinking that I am going to die.

2. What are the treatment options for cervical cancer?

   R: I don't know

Probe: Which treatment did your healthcare provider recommend for you?

R: I don't know

3. What specific conditions you were given to follow during your treatment for cervical cancer?

   R: I dint not given any specific conditions to follow. My fellow patients told me that I will have to do this and that and expected that the doctor will inform me but unfortunately he did not.

Probe: Were they clear and easy to understand?

4. What did your healthcare provider explain to you regarding your treatment regimen?

   R: He told me that my external beam radiotherapy will go on for 25 days and then brachytherapy for 3 weeks, after that I will the doctor for follow-up appointments

Probe: How long would it take you to complete your planned treatments?

Probe: How frequently were you supposed to receive your treatments?

5. What challenges did you face in adhering to your treatment for cervical cancer?
   R: Some disturbances that require moving from one office to another due to unclear information about paying consultation fees.

Probe: How have you managed these challenges?

6. What is your understanding of the importance of adherence to treatment plans for cervical cancer?

   R: I adhere to my treatments, I feeling better now through I feel some stomach pain sometimes. I shared with my doctor, and we are proceeding well.

7. During your attendance for cancer care in Bugando Oncology Clinic, have you ever received teachings or materials (pictures, flyers etc.) to help improve your adherence?

   R: Yes I have received teachings and flyers

8. Is there anything else you would like to share about your experience with cervical cancer?

   R: I don't have

9. What questions or concerns do you have about cervical cancer and its management?

   R: Since the research about cervical cancer have begun, haven't they find out what cause cervical cancer?

Appendix ii (a): Interview Guide - English Version

“For non-adherent cervical cancer patients attending Bugando Oncology Clinic”

Identification number – BMC Onco 02 Date for an in-depth interview – 30/08/2023

A. Participant’s Socio-demographic Information

1. Age (years) – 47
2. Highest education level reached – Standard 7
3. Occupation – Farming cassava
4. Religious denomination – Christianity Mennonite
5. Marital status – Widow
6. Residence (district, region) – Kirogwe, Rorya, Mara

B. Exploring of healthcare-seeking behaviors

1. Nowadays there are many health problems affecting people of the lake zone, and cancer is on the increase, mention types of cancers?

   R: Cervical cancer, Breast cancer

Probe: (If cervical cancer is not mentioned) How about cervical cancer?

2. What are the symptoms of cervical cancer?

   R: I had post coital bleeding, smelling discharge, waist and abdominal pain, my private parts were itching and burning sensation.

Probe: What symptoms or concerns led you to seek healthcare for your cervical cancer?

R: I had severe pain, bleeding and bad smelling. As woman it is totally uncomfortable to have smelling discharge, it was difficult to sit in the church as people saw me smelling, and when I

stand up my clothes looked wet with blood. When church fellows come to my home, I told them I am smelling badly.

3. Tell me any instances where you tried to treat yourself for cervical cancer or its symptoms.

Probe: What led you to believe that self-treatment was necessary or preferable?

4. What other alternative or complementary treatments have you sought?

   R: I was given the traditional medicine locally called “nyanjugo”, which is administered by drinking and also by sitting in the mixture of that medicine and water.

Probe: Please, explain your experiences with these treatments.

R: I dint get any relief, and the pain was much severe so I decided to go to Bugando

Probe: How did you make a decision about which treatment to pursue?

R: I went to a dispensary but did not know what I was suffering from, then I went to a hospital in Kenya, where they told me to be referred to another hospital in Kenya, and they gave me a painkiller drug. I told them that hospital is very far so I decided to go to Shirati hospital after a week. At the Shirati hospital, a nurse screened me and said that the disease looks like cervical cancer so she had to refer me to Bugando Medical Centre. I told them I don’t know anyone at Bugando, then who will help me and where will I get the money as I have neither mother nor child to rely on. They told me to go looking for money and if I will get enough money I should return to have a referral letter. I told my church fellows and they contributed me some money, and I add up mine, thereafter I went to Shirati hospital where they gave me a referral letter to Bugando.

5. What barriers or challenges have you faced in seeking healthcare for your cervical cancer?

   R: Money to go to Bugando as we always heard that to go to Bugando someone must have money.

Probe: How have you managed to overcome those challenges?

R: My church fellows encouraged and contributed me some money and I add up mine.

6. Describe sociocultural factors that have influenced your decision-making for cancer care.

Probe: What specific misconceptions you have heard about cervical cancer treatment?

R: We always hear that "The disease that cannot be treated at Shirati Hospital cannot even be treated at Bugando. Going to Bugando will lead to only dearth." I just said even if I die at Bugando, my church fellows will come to take me.

7. What advice would you give to other women who may be experiencing symptoms or concerns related to cervical cancer?

   R: I will advise her to go to Bugando.

8. Is there anything else you would like to share about your experience with seeking healthcare for your cervical cancer?

   R: My treatment is going well but when we got to Bugando, the doctor told us to find money, Tsh. 328,000/=. We then went back home as the machine went broke. We got back to Bugando after the machine was fixed. Then another doctor told us that treatment for cervical cancer is free, then after a while we were told that money is needed to consult a doctor Tsh 5,000, when you are going for blood test Tsh. 17,000/=, to have chemotherapy money is needed, for brachytherapy money is needed more than Tsh. 300,000/=. How is it for me as I am totally broke, sometimes I failed to get chemotherapy as I did have money but the day after, they helped me by providing free chemotherapy to me.

C. Identifying factors influencing non-adherence

1. What were your initial thoughts and feelings when you were diagnosed with cervical cancer?

R: The nurse told me I are you ready to receive your diagnosis, are you alone? I told her that the one escorted me to Bugando is not my relative but just my neighbor, she is someone who is helping me as she works in Mwanza, so I am ready to get my diagnosis alone. The nurse told me that I had cervical cancer, I had some thinking, and she said sorry to me, I then felt some relief. I asked her, is it treatable, she said yes, it is treatable. Then I felt some hope that the cervical cancer is treatable.

2. What are the treatment options for cervical cancer?

Probe: Which treatment did your healthcare provider recommend for you?

R: I was not informed by the healthcare provider. I met a fellow tribal women, who told me she had the same disease, and she is going on with the treatments. She told me that treatment is not a syringe, it a machine. U just slept on the bed and the machine deliver the treatment.

3. What specific conditions you were given to follow during your treatment for cervical cancer?

   R: Yes, I was told to drink plenty of water, to consider hygiene as carrying underwear, and I carried it with me.

Probe: Were they clear and easy to understand?

R: Yes, I understood the conditions

4. What did your healthcare provider explain to you regarding your treatment regimen?

Probe: How long would it take you to complete your planned treatments?

R: I was told that, I will receive radiotherapy for 25 days and then after I will go on with brachytherapy for three weeks.

Probe: How frequently were you supposed to receive your treatments?

R: Once a week for brachytherapy

5. What challenges did you face in adhering to your treatment for cervical cancer?

R: I did not get any challenge other than machine breakdown, and failed to have timely chemotherapy because I didn't have money.
Probe: How have you managed these challenges?

6. What is your understanding of the importance of adherence to treatment plans for cervical cancer?

   R: I felt much better, no pain and the severe bleeding turned to be little, and no smelling.

7. During your attendance for cancer care in Bugando Oncology Clinic, have you ever received teachings or materials (pictures, flyers etc.) to help improve your adherence?

   R: Yes, I received pictures and we had teachings about issues like drinking water, hygiene and the time to come for treatment.

8. Is there anything else you would like to share about your experience with cervical cancer?

9. R: No

10. What questions or concerns do you have about cervical cancer and its management?

    R: Just want to ask that, after finishing treatments, do we just stay at home?

    R: And also, does the treatments also stop my monthly menses?

Appendix ii (a): Interview Guide - English Version

"For non-adherent cervical cancer patients attending Bugando Oncology Clinic"

Identification number – BMC Onco 03 Date for an in-depth interview – 02/09/2023

A. Participant's Socio-demographic Information

1. Age (years) – 39
2. Highest education level reached – Secondary Form II
3. Occupation – Entrepreneur
4. Religious denomination – Muslim
5. Marital status – Divorced
6. Residence (district, region) – Buhongwa, Nyamagana, Mwanza.

B. Exploring of healthcare-seeking behaviors

1. Nowadays there are many health problems affecting people of the lake zone, and cancer is on the increase, mention types of cancers?

   R: Throat cancer, cervical cancer, prostate cancer

Probe: (If cervical cancer is not mentioned) How about cervical cancer?

2. What are the symptoms of cervical cancer?

   R: Nonstop bleeding

Probe: What symptoms or concerns led you to seek healthcare for your cervical cancer?

R: I had nonstop bleeding from December 2022 to June 2023 where I had hysterectomy at Bugando. I went to Nyamagana Hospital, Butimba where they diagnosed me with Cervical cancer and they referred me to Bugando where I had some tests and underwent hysterectomy on 02 May 2023. Then my doctor referred me to Oncology Department, where the doctor read

my history and told me about treatments in the Oncology Department. Unfortunately, I was told that the machine is broke, so I will have to wait for a month to start radiotherapy treatment.

3. Tell me any instances where you tried to treat yourself for cervical cancer or its symptoms.

   R: I just went to private hospital where I was sometimes told that I have PID, and sometimes I was told I had UTI that has reached 60. When I had heavy bleeding, and sometimes severe anemia that made me fall down, I told my daughter that lets go to Butimba, the government hospital, as I don't get any better in the private hospitals.

Probe: What led you to believe that self-treatment was necessary or preferable?

4. What other alternative or complementary treatments have you sought?

R: Just have some medicine. I didn't had faith to go to traditional healers because even my grandmother had have cervical cancer. I just went to my auntie who cared about my grandmother and told her about my condition. She told me that my symptoms resemble those of my grandmother, she encouraged me to go to hospital, and that my grandmother underwent hysterectomy and her life went on well and died from other illness. My auntie encouraged and insisted me, then I stopped crying, and then I went to hospital.

Probe: Please, explain your experiences with these treatments.

Probe: How did you make a decision about which treatment to pursue?

5. What barriers or challenges have you faced in seeking healthcare for your cervical cancer?

   R: No, I didn't. Just I was diagnosed with diabetes, and anemia. Also it was difficult to afford the treatments as cancer treatments are expensive.

Probe: How have you managed to overcome those challenges?

6. Describe sociocultural factors that have influenced your decision-making for cancer care.

R: It was just my auntie and my personal experience as my grandmother used to tell me about management of her cervical cancer.

Probe: What specific misconceptions you have heard about cervical cancer treatment?

7. What advice would you give to other women who may be experiencing symptoms or concerns related to cervical cancer?

   R: I will just advise her to come straight to the hospital, as you can see I am now doing well to the extent of walking around without wearing an underwear.

8. Is there anything else you would like to share about your experience with seeking healthcare for your cervical cancer?

   R: I just rely on God, and trust the doctor with his treatments and advises.

C. Identifying factors influencing non-adherence

1. What were your initial thoughts and feelings when you were diagnosed with cervical cancer?

   R: Firstly, I had a challenge with my marriage, I got divorced without any genuine reason. I kept it aside and told God I need my health as I have three children, one is in standard five, the second one is in form two, and the third one is a dependent, has completed university education and not yet got an employment, just struggle with life. Both of them are girls. Also I don't have a mother, and my father has another family. Then who will help me with my treatments? I was born alone, my mother died. So I was very much stressed. My treatment looked difficult, my life is difficult. By then, I was a mother and a father, and needed to hustle. I thank God that by the time my marriage started to be a challenge, I was doing business, I lived with budgeting. I borrowed some money from the lending groups to build a house. Now that I am in my house, I don't pay rent but feeding my children, paying the schools fees and affording my treatments I must cope with the situation.

The respondent cried and the researcher assistant comforted her and apologized for reminding the respondent about her previous hardships.

2. What are the treatment options for cervical cancer?

Probe: Which treatment did your healthcare provider recommend for you?

R: My doctor informed me that, I will treated by the external beam radiotherapy, brachytherapy and chemotherapy

3. What specific conditions you were given to follow during your treatment for cervical cancer?

   R: To eat well, to rest, to avoid stress, to drink plenty of water, to eat food that increase my blood.

Probe: What were the challenges you faced to cope with those conditions.

R: I didn’t faced any challenges, as since the time I was admitted with diabetes, I got familiar with these conditions so as to control my blood sugar level. So to me it was fine.

4. What did your healthcare provider explain to you regarding your treatment regimen?

   R: I was told that I will have four cycles of chemotherapy and radiotherapy both external and internal.

Probe: How long would it take you to complete your planned treatments?

Probe: How frequently were you supposed to receive your treatments?

R: Chemotherapy once a week, on Mondays.

5. What challenges did you face in adhering to your treatment for cervical cancer?
   R: I didn’t get any, I adhere so as I get healed and continue to care for my children.

Probe: How have you managed these challenges?

6. What is your understanding of the importance of adherence to treatment plans for cervical cancer?

   R: Honestly, I feel better, although I had some sores and fatigue because of radiation.

7. During your attendance for cancer care in Bugando Oncology Clinic, have you ever received teachings or materials (pictures, flyers etc.) to help improve your adherence?

   R: For me, when I didn't have chemotherapy, I went to Bugando at 04:00 pm. Coming early was a bit challenge as I stay away from Bugando, so usually I ended up got number 60 out of 80. Waiting until my number is called for treatment was difficult for me as where shall I eat, how can I survive the day while my life is this difficult. So I attended the teachings only in the first week. After realizing that the teaching schedule is not friendly for me, I decided to come at the evening, so I can eat at home, and have an enough rest before coming for radiotherapy.
8. Is there anything else you would like to share about your experience with cervical cancer?
9. What questions or concerns do you have about cervical cancer and its management?

   R: Just want to ask, after I have completed all the treatments, will I be healed completely?

Appendix ii (a): Interview Guide - English Version

"For non-adherent cervical cancer patients attending Bugando Oncology Clinic"

Identification number – BMC Onco 04 Date for an in-depth interview – 30/08/2023

A. Participant's Socio-demographic Information

1. Age (years) – 61
2. Highest education level reached – No formal education
3. Occupation – Peasant
4. Religious denomination – No religion
5. Marital status – Widow
6. Residence (district, region) – Rorya, Mara

B. Exploring of healthcare-seeking behaviors

1. Nowadays there are many health problems affecting people of the lake zone, and cancer is on the increase, mention types of cancers?

   R: None

Probe: (If cervical cancer is not mentioned) How about cervical cancer?

2. What are the symptoms of cervical cancer?

   R: Post-menopausal bleeding, stomach pain

Probe: What symptoms or concerns led you to seek healthcare for your cervical cancer?

3. Tell me any instances where you tried to treat yourself for cervical cancer or its symptoms.

Probe: What led you to believe that self-treatment was necessary or preferable?

4. What other alternative or complementary treatments have you sought?

   R: Traditional medicine

Probe: Please, explain your experiences with these treatments.

R: I used traditional medicine for 2 days

Probe: How did you make a decision about which treatment to pursue?

5. What barriers or challenges have you faced in seeking healthcare for your cervical cancer?

   R: I didn't have money for cancer services

Probe: How have you managed to overcome those challenges?

R: My daughter supported me financially

6. Describe sociocultural factors that have influenced your decision-making for cancer care.

   R: My daughter convinced me to go to hospital

Probe: What specific misconceptions you have heard about cervical cancer treatment?

R: Cervical cancer is not treatable, Radiotherapy involve the use of fire to burn a body part

7. What advice would you give to other women who may be experiencing symptoms or concerns related to cervical cancer?

   R: I will advise her that cervical cancer is treatable, she has to go the hospital. I will testify using myself as an example of someone who have healed from cervical cancer.

8. Is there anything else you would like to share about your experience with seeking healthcare for your cervical cancer?

C. Identifying factors influencing non-adherence

1. What were your initial thoughts and feelings when you were diagnosed with cervical cancer?

   R: I just felt as a normal thing, and believed that God will help me.

2. What are the treatment options for cervical cancer?

Probe: Which treatment did your healthcare provider recommend for you?

R: Radiotherapy

3. What specific conditions you were given to follow during your treatment for cervical cancer?

   R: No specific conditions

Probe: Were they clear and easy to understand?

4. What did your healthcare provider explain to you regarding your treatment regimen?

   R: To eat a lot of fruits, to eat well and enough

Probe: How long would it take you to complete your planned treatments?

Probe: How frequently were you supposed to receive your treatments?

5. What challenges did you face in adhering to your treatment for cervical cancer?
   R: machine breakdown

Probe: How have you managed these challenges?

6. What is your understanding of the importance of adherence to treatment plans for cervical cancer?

   R: I don't bleed any more

7. During your attendance for cancer care in Bugando Oncology Clinic, have you ever received teachings or materials (pictures, flyers etc.) to help improve your adherence?

   R: I once received flyers, and teachings on time to come for treatment

8. Is there anything else you would like to share about your experience with cervical cancer?

9. What questions or concerns do you have about cervical cancer and its management?

The respondents has trouble in understanding Swahili.

Appendix ii (a): Interview Guide - English Version

"For non-adherent cervical cancer patients attending Bugando Oncology Clinic"

Identification number – BMC Onco 05   Date for an in-depth interview – 05/08/2025

A. Participant's Socio-demographic Information

1. Age (years) – 50
2. Highest education level reached – No formal education
3. Occupation – Peasant
4. Religious denomination – Muslim
5. Marital status – Married
6. Residence (district, region) – Malolo, Tabora Municipal

B. Exploring of healthcare-seeking behaviors

1. Nowadays there are many health problems affecting people of the lake zone, and cancer is on the increase, mention types of cancers?

   R: I don't know

Probe: (If cervical cancer is not mentioned) How about cervical cancer?

2. What are the symptoms of cervical cancer?

   R: Post coital bleeding

Probe: What symptoms or concerns led you to seek healthcare for your cervical cancer?

R: Severe bleeding, fatigue and waist pain

3. Tell me any instances where you tried to treat yourself for cervical cancer or its symptoms.

   No, I didn't try.

Probe: What led you to believe that self-treatment was necessary or preferable?

4. What other alternative or complementary treatments have you sought?

   R: I went to traditional healers but the symptoms were worsen

Probe: Please, explain your experiences with these treatments.

R: No I don't know

Probe: How did you make a decision about which treatment to pursue?

R: There was a screening camp in my village, I went there where there screened me and look like I have a tumor. I was told to go to the hospital but I didn't have money so I didn't go. After a month, I had severe bleeding, I went back to the hospital and they told me I am a suspicious of cervical cancer. They asked me to pay Tsh. 100,000 for biopsy test. I didn't have the money and told them let me find it. After a month I went back, and they told me to go to Malolo Hospital, where they told me that I have cervical cancer.

5. What barriers or challenges have you faced in seeking healthcare for your cervical cancer?

   R: I thank God, I didn't get any challenge rather that machine breakdown

Probe: How have you managed to overcome those challenges?

6. Describe sociocultural factors that have influenced your decision-making for cancer care.

   R: It was just the symptoms of my disease, and family supported me to go to the hospital.

Probe: What specific misconceptions you have heard about cervical cancer treatment?

7. What advice would you give to other women who may be experiencing symptoms or concerns related to cervical cancer?

   R: I will advise them to check on possible cervical cancer symptoms like heavy bleeding, smelling discharge, post coital bleeding, fatigue, back pain

8. Is there anything else you would like to share about your experience with seeking healthcare for your cervical cancer?

R: Just need your cooperation so as I can get better treatment and be healed

C. Identifying factors influencing non-adherence

1. What were your initial thoughts and feelings when you were diagnosed with cervical cancer?

   R: My heart was beating very fast, I didn't have peace of mind, as day's back we used to know that someone with cancer will die very soon, so I felt like my days are numbered

2. What are the treatment options for cervical cancer?

   R: I don't know, I just get treatments

Probe: Which treatment did your healthcare provider recommend for you?

3. What specific conditions you were given to follow during your treatment for cervical cancer?

   R: No, I just here some patients say this and others say that, but I didn't get informed

Probe: Were they clear and easy to understand?

4. What did your healthcare provider explain to you regarding your treatment regimen?

   R: I was told that external beam radiotherapy are 25, and brachytherapy are three.

Probe: How long would it take you to complete your planned treatments?

Probe: How frequently were you supposed to receive your treatments?

5. What challenges did you face in adhering to your treatment for cervical cancer?
   R: I faced challenges due to being unfamiliar and I had no one to help me

Probe: How have you managed these challenges?

R: As I had the problems, I opted to be patient

6. What is your understanding of the importance of adherence to treatment plans for cervical cancer?

   R: I have a relief of my symptoms, but still have some pain

7. During your attendance for cancer care in Bugando Oncology Clinic, have you ever received teachings or materials (pictures, flyers etc.) to help improve your adherence?

R: We have given some flyers, but was very few so I once read them. I always attend the teachings.

8. Is there anything else you would like to share about your experience with cervical cancer?
9. What questions or concerns do you have about cervical cancer and its management?

   R: What is the late stage of cancer?

   R: What if you have another disease, will cancer be healed and the other disease remain?

Appendix ii (a): Interview Guide - English Version

"For non-adherent cervical cancer patients attending Bugando Oncology Clinic"

Identification number – BMC Onco 6 Date for an in-depth interview – 05/09/2023

A. Participant's Socio-demographic Information

1. Age (years) – 52
2. Highest education level reached – Standard Seven
3. Occupation – Business woman
4. Religious denomination – Muslim
5. Marital status – Separated
6. Residence (district, region) – Igunga, Tabora

B. Exploring of healthcare-seeking behaviors

1. Nowadays there are many health problems affecting people of the lake zone, and cancer is on the increase, mention types of cancers?

   R: Cervical cancer, prostate state, throat cancer, lung cancer, leukemia, brain cancer, skin cancer, bladder cancer, colon cancer, anorectal cancer

Probe: (If cervical cancer is not mentioned) How about cervical cancer?

2. What are the symptoms of cervical cancer?

   R: Postmenopausal bleeding, a tumor protruding out of my vagina, vaginal discharge

Probe: What symptoms or concerns led you to seek healthcare for your cervical cancer?

3. Tell me any instances where you tried to treat yourself for cervical cancer or its symptoms.

   R: I tried to use traditional medicine that treat worms

Probe: What led you to believe that self-treatment was necessary or preferable?

4. What other alternative or complementary treatments have you sought?

   R: No I didn’t

Probe: Please, explain your experiences with these treatments.

Probe: How did you make a decision about which treatment to pursue?

5. What barriers or challenges have you faced in seeking healthcare for your cervical cancer?

   R: I didn’t get any challenge. My daughter supported me financially

Probe: How have you managed to overcome those challenges?

6. Describe sociocultural factors that have influenced your decision-making for cancer care.

   R: I usually like to go straight to the hospital when I have a health issue

Probe: What specific misconceptions you have heard about cervical cancer treatment?

7. What advice would you give to other women who may be experiencing symptoms or concerns related to cervical cancer?

   R: I have started to tell some patients with cancer symptoms to just go to the hospital to check for cancer.

8. Is there anything else you would like to share about your experience with seeking healthcare for your cervical cancer?

C. Identifying factors influencing non-adherence

1. What were your initial thoughts and feelings when you were diagnosed with cervical cancer?

   R: My daughter didn’t tell me because she know that I have low blood pressure. The doctor told me that I should not be afraid because I have pressure, I told him to just tell me as diseases are part of everyday life. After he told me I have cancer, I just wonder, how did I have it? I just believed that God will help me. I went to Bugando and see children with eye cancer, I felt

myself healed and I asked myself if children like these can have cancer, who am I not to have cancer? From that day I saw cancer as normal disease.

2. What are the treatment options for cervical cancer?

   R: Not informed at all, just told to go on with radiotherapy

Probe: Which treatment did your healthcare provider recommend for you?

3. What specific conditions you were given to follow during your treatment for cervical cancer?

   R: I was told to eat well

Probe: Were they clear and easy to understand?

4. What did your healthcare provider explain to you regarding your treatment regimen?

   R: I was told that I will receive 25 radiotherapy and 5 chemotherapy

Probe: How long would it take you to complete your planned treatments?

Probe: How frequently were you supposed to receive your treatments?

5. What challenges did you face in adhering to your treatment for cervical cancer?

   R: First weeks we used to eat at the cafeteria in the hospital but later on we find it financially difficult.

   R: Still stomach pain, and I have diarrhea but everything else is fine.

Probe: How have you managed these challenges?

6. What is your understanding of the importance of adherence to treatment plans for cervical cancer?

   R: I was improving

7. During your attendance for cancer care in Bugando Oncology Clinic, have you ever received teachings or materials (pictures, flyers etc.) to help improve your adherence?

   R: Staying here at Machinjioni made me difficult to attend the teachings as I always went late to Bugando due to transport difficulties. I just attended the teaching once.

8. Is there anything else you would like to share about your experience with cervical cancer?

   I used to rub my body parts that had some pain to find relief. I also had some ulcer as the radiotherapy side effects

9. What questions or concerns do you have about cervical cancer and its management?

   R: Since I had anesthetic surgery a month before brachytherapy which also uses anesthesia, is it safe?

Appendix ii (a): Interview Guide - English Version

"For non-adherent cervical cancer patients attending Bugando Oncology Clinic"

Identification number – BMC Onco 07 Date for an in-depth interview – 02/09/2023

A. Participant's Socio-demographic Information

1. Age (years) – 60
2. Highest education level reached – Standard seven
3. Occupation – Mkulima
4. Religious denomination – Roman Catholic
5. Marital status – Widow
6. Residence (district, region) – Nyakato, Ilemela, Mwanza.

B. Exploring of healthcare-seeking behaviors

1. Nowadays there are many health problems affecting people of the lake zone, and cancer is on the increase, mention types of cancers?

   R: Skin cancer, cervical cancer, throat cancer

Probe: (If cervical cancer is not mentioned) How about cervical cancer?

2. What are the symptoms of cervical cancer?

   R: Back pain, one leg swelling, waist pain, lower abdominal pain, sensing a tumor

Probe: What symptoms or concerns led you to seek healthcare for your cervical cancer?

3. Tell me any instances where you tried to treat yourself for cervical cancer or its symptoms.

   I rub my back when I had go and return back pain

Probe: What led you to believe that self-treatment was necessary or preferable?

4. What other alternative or complementary treatments have you sought?

R: During the period of one month waiting for my final diagnosis at Bugando, I shared this information with my friends, who provided me the many traditional medicine but I didn't find any relief.

Probe: Please, explain your experiences with these treatments.

Probe: How did you make a decision about which treatment to pursue?

5. What barriers or challenges have you faced in seeking healthcare for your cervical cancer?

   R: My early visit after onset of symptoms at the hospital I was told that I have UTI and typhoid, and given the respective medicines but I found no relief.

   R: I was supposed to pay Tsh. 50,000 but didn't have the money, so he told me to go until I got the money. Then I came the next day, and told that I should have to wait for one month to have a diagnosis.

Probe: How have you managed to overcome those challenges?

R: I went to Sekou Toure hospital where the doctors referred me to Bugando.

6. Describe sociocultural factors that have influenced your decision-making for cancer care.

   R: When you are sick, you just can sit and wait, u will find the relief.

Probe: What specific misconceptions you have heard about cervical cancer treatment?

7. What advice would you give to other women who may be experiencing symptoms or concerns related to cervical cancer?

   R: I will advise to not delay in seeking hospital cancer care

8. Is there anything else you would like to share about your experience with seeking healthcare for your cervical cancer?

9. R: I don't have

C. Identifying factors influencing non-adherence

1. What were your initial thoughts and feelings when you were diagnosed with cervical cancer?

   R: I thought that this disease is not treatable, and I have got it, therefore I don't know about my future. But after sometime when I got home, I just said to myself "No one know how s/he will die, this disease is above my capability but only God know where I can get the treatments.

2. What are the treatment options for cervical cancer?

   R: I have gone through EB Radiotherapy, chemotherapy and brachytherapy

Probe: Which treatment did your healthcare provider recommend for you?

3. What specific conditions you were given to follow during your treatment for cervical cancer?

   R: We were told to eat well, a lot of fruits and plenty of water.

Probe: Were they clear and easy to understand?

4. What did your healthcare provider explain to you regarding your treatment regimen?

   R: I was told that my radiotherapy will take five weeks that is 25 days

Probe: How long would it take you to complete your planned treatments?

Probe: How frequently were you supposed to receive your treatments?

5. What challenges did you face in adhering to your treatment for cervical cancer?
   R: My radiotherapy was delayed because the machine was broke.
   R: As you receive treatment, you find yourself with no food appetite.

Probe: How have you managed these challenges?

6. What is your understanding of the importance of adherence to treatment plans for cervical cancer?

   R: I see my health is improving, though I have some lower abdominal pain

7. During your attendance for cancer care in Bugando Oncology Clinic, have you ever received teachings or materials (pictures, flyers etc.) to help improve your adherence?

   R: No, not at all

8. Is there anything else you would like to share about your experience with cervical cancer?

9. What questions or concerns do you have about cervical cancer and its management?

Appendix ii (a): Interview Guide - English Version

"For non-adherent cervical cancer patients attending Bugando Oncology Clinic"

Identification number – BMC Onco 08 Date for an in-depth interview – 04/09/2023

A. Participant's Socio-demographic Information

1. Age (years) – 57
2. Highest education level reached – Standard three
3. Occupation – Peasant
4. Religious denomination – Roman Catholic
5. Marital status – Divorced
6. Residence (district, region) – Maswa, Simiyu

B. Exploring of healthcare-seeking behaviors

1. Nowadays there are many health problems affecting people of the lake zone, and cancer is on the increase, mention types of cancers?

   R: Cervical cancer

Probe: (If cervical cancer is not mentioned) How about cervical cancer?

2. What are the symptoms of cervical cancer?

   R: I had severe bleeding and a tumor, ulcers in private parts, vaginal discharge

Probe: What symptoms or concerns led you to seek healthcare for your cervical cancer?

3. Tell me any instances where you tried to treat yourself for cervical cancer or its symptoms.

   R: I was just buying UTI medicines, but I didn't find any relief.

Probe: What led you to believe that self-treatment was necessary or preferable?

4. What other alternative or complementary treatments have you sought?

Probe: Please, explain your experiences with these treatments.

Probe: How did you make a decision about which treatment to pursue?

5. What barriers or challenges have you faced in seeking healthcare for your cervical cancer?

   R: AT Bugando, I were told to wait for two weeks to start radiotherapy as the machine has reached its maximum capacity.

Probe: How have you managed to overcome those challenges?

6. Describe sociocultural factors that have influenced your decision-making for cancer care.

   R: The family decided that I have to be taken to the hospital. Therefore we went to district hospital, where they told my son that they suspect me to have cervical cancer and referred me to Bugando.

Probe: What specific misconceptions you have heard about cervical cancer treatment?

7. What advice would you give to other women who may be experiencing symptoms or concerns related to cervical cancer?

8. Is there anything else you would like to share about your experience with seeking healthcare for your cervical cancer?

C. Identifying factors influencing non-adherence

1. What were your initial thoughts and feelings when you were diagnosed with cervical cancer?

   R: I felt to go to hospital

2. What are the treatment options for cervical cancer?

   R: Radiotherapy

Probe: Which treatment did your healthcare provider recommend for you?

R: Radiotherapy

3. What specific conditions you were given to follow during your treatment for cervical cancer?

R: To consult a doctor on Thursdays, to do some laboratory tests before coming for chemotherapy on Mondays.

R: To I was told to eat vegetables and to drink plenty of water

Probe: Were they clear and easy to understand?

4. What did your healthcare provider explain to you regarding your treatment regimen?

   R: I was told that my treatment will take 25 days to complete

Probe: How long would it take you to complete your planned treatments?

Probe: How frequently were you supposed to receive your treatments?

5. What challenges did you face in adhering to your treatment for cervical cancer?
   R: The disease make me be like a disabled.

Probe: How have you managed these challenges?

6. What is your understanding of the importance of adherence to treatment plans for cervical cancer?

   R: I must adhere to treatment plans so I went for treatment daily

7. During your attendance for cancer care in Bugando Oncology Clinic, have you ever received teachings or materials (pictures, flyers etc.) to help improve your adherence?

   R: I didn’t attend the teachings or given any flyers.

8. Is there anything else you would like to share about your experience with cervical cancer?

9. What questions or concerns do you have about cervical cancer and its management?

   R: I want to know why I still have vaginal discharge?

Appendix ii (a): Interview Guide - English Version

"For non-adherent cervical cancer patients attending Bugando Oncology Clinic"

Identification number – BMC Onco 09 Date for an in-depth interview – 03/09/2023

A. Participant's Socio-demographic Information

1. Age (years) – 58
2. Highest education level reached – Standard seven
3. Occupation – Peasant
4. Religious denomination – Roman Catholic
5. Marital status – Married
6. Residence (district, region) – Shinyanga

B. Exploring of healthcare-seeking behaviors

1. Nowadays there are many health problems affecting people of the lake zone, and cancer is on the increase, mention types of cancers?

   R: Cervical cancer, neck tumors, throat cancers, eye cancers

Probe: (If cervical cancer is not mentioned) How about cervical cancer?

2. What are the symptoms of cervical cancer?

   R: I was having bleeding, vaginal discharge

Probe: What symptoms or concerns led you to seek healthcare for your cervical cancer?

3. Tell me any instances where you tried to treat yourself for cervical cancer or its symptoms.

R: I used medicine from the pharmacy

Probe: What led you to believe that self-treatment was necessary or preferable?

4. What other alternative or complementary treatments have you sought?

I used some traditional medicine that worsen my situation, thereafter I went to Shinyanga Hospital.

Probe: Please, explain your experiences with these treatments.

Probe: How did you make a decision about which treatment to pursue?

R: My relatives advised me to go to the hospital and check for cervical cancer

5. What barriers or challenges have you faced in seeking healthcare for your cervical cancer?

   R: I was being treated as I am having UTI, typhoid or fungus

Probe: How have you managed to overcome those challenges?

6. Describe sociocultural factors that have influenced your decision-making for cancer care.

Probe: What specific misconceptions you have heard about cervical cancer treatment?

7. What advice would you give to other women who may be experiencing symptoms or concerns related to cervical cancer?

   R: Just she has to go for cervical cancer screening

8. Is there anything else you would like to share about your experience with seeking healthcare for your cervical cancer?

   R: I would like to share the news that cervical cancer is treatable and we are healed. The way I used to suffer, I don't suffer that way anymore.

C. Identifying factors influencing non-adherence

1. What were your initial thoughts and feelings when you were diagnosed with cervical cancer?

   R: I didn't think anything rather than to go for the treatment so that I can be healed.

2. What are the treatment options for cervical cancer?

Probe: Which treatment did your healthcare provider recommend for you?

R: I was told that I have cervical cancer so I should go for radiotherapy

3. What specific conditions you were given to follow during your treatment for cervical cancer?

   R: I was told not to be under the sun, not to be around fire. I find it difficult to have food appetite.

Probe: Were they clear and easy to understand?

4. What did your healthcare provider explain to you regarding your treatment regimen?

   R: I was told that my radiotherapy will take 25 days, and I have to go for laboratory tests on Thursdays and get the results on Friday, so as to have chemotherapy on Mondays.

Probe: How long would it take you to complete your planned treatments?

Probe: How frequently were you supposed to receive your treatments?

5. What challenges did you face in adhering to your treatment for cervical cancer?
   R: My body was feeling not well, and I had no food appetite

Probe: How have you managed these challenges?

6. What is your understanding of the importance of adherence to treatment plans for cervical cancer?

   R: Adhering to treatment is important to control the symptoms and get healed.

7. During your attendance for cancer care in Bugando Oncology Clinic, have you ever received teachings or materials (pictures, flyers etc.) to help improve your adherence?

   R: I attended the teachings but I didn't get the flyers as only few copies were given and I didn't get one. Please if you have one, give me.

8. Is there anything else you would like to share about your experience with cervical cancer?

9. What questions or concerns do you have about cervical cancer and its management?

   R: what causes cancer?

Appendix ii (a): Interview Guide - English Version

"For non-adherent cervical cancer patients attending Bugando Oncology Clinic"

Identification number – BMC Onco 10 Date for an in-depth interview – 02/09/2023

A. Participant's Socio-demographic Information

1. Age (years) – 48
2. Highest education level reached – No formal education
3. Occupation – Peasant
4. Religious denomination – Baptist
5. Marital status – Married
6. Residence (district, region) – Nyakato, Ilemela, Mwanza

B. Exploring of healthcare-seeking behaviors

1. Nowadays there are many health problems affecting people of the lake zone, and cancer is on the increase, mention types of cancers?

   R: Cervical cancer, throat cancer, breast cancer, lung cancer

Probe: (If cervical cancer is not mentioned) How about cervical cancer?

2. What are the symptoms of cervical cancer?

   R: Lower abdominal pain, post coital bleeding

Probe: What symptoms or concerns led you to seek healthcare for your cervical cancer?

R: Post coital bleeding

3. Tell me any instances where you tried to treat yourself for cervical cancer or its symptoms.

   R: I didn't

Probe: What led you to believe that self-treatment was necessary or preferable?

4. What other alternative or complementary treatments have you sought?

Probe: Please, explain your experiences with these treatments.

Probe: How did you make a decision about which treatment to pursue?

5. What barriers or challenges have you faced in seeking healthcare for your cervical cancer?

   I had no money Tsh. 80,000 for cervical cancer diagnosis at Bugando so they took a sample and told me to find the money so as to get my diagnosis. I went back home and felt more severe bleeding thus I went to Buzuruga health center, they admitted me and told me that I have bacteria in uterus. They told me to buy medicine for Tsh. 80,000 but I only afforded half a dose. The situation kept worsening and I referred to Sekou Toure, where the money was also a challenge, but finally we managed to get the money and diagnosed with cervical cancer. My husband went for my diagnosis report to Bugando and when we show it to Sekou Toure they referred me Bugando for cervical cancer treatment. So my biggest challenge was financial barriers.

   Probe: How have you managed to overcome those challenges?

   R: My family side supported me, and my husband didn't have money so it lead to family misunderstanding.

6. Describe sociocultural factors that have influenced your decision-making for cancer care.

   R: Only the symptoms forced me to look for the healthcare

Probe: What specific misconceptions you have heard about cervical cancer treatment?

7. What advice would you give to other women who may be experiencing symptoms or concerns related to cervical cancer?

   R: I advise them to go to Bugando, for cervical cancer diagnosis as it is free service.

8. Is there anything else you would like to share about your experience with seeking healthcare for your cervical cancer?

C. Identifying factors influencing non-adherence

1. What were your initial thoughts and feelings when you were diagnosed with cervical cancer?

   R: I thought about how we will afford the treatments as my family is not well financially. But I thank God.

2. What are the treatment options for cervical cancer?

Probe: Which treatment did your healthcare provider recommend for you?

R: I was planned to have radiotherapy for 25 days and then brachytherapy

3. What specific conditions you were given to follow during your treatment for cervical cancer?

   R: To consider eating well, consuming fruits and vegetable, and drinking plenty of water

Probe: Were they clear and easy to understand?

4. What did your healthcare provider explain to you regarding your treatment regimen?

Probe: How long would it take you to complete your planned treatments?

Probe: How frequently were you supposed to receive your treatments?

5. What challenges did you face in adhering to your treatment for cervical cancer?
   R: challenges rose when we had no money

Probe: How have you managed these challenges?

6. What is your understanding of the importance of adherence to treatment plans for cervical cancer?

   R: My situation had improved, although I still have some discomfort but not as it was before, no more bleeding.

7. During your attendance for cancer care in Bugando Oncology Clinic, have you ever received teachings or materials (pictures, flyers etc.) to help improve your adherence?

R: Yes, we always receive teachings

8. Is there anything else you would like to share about your experience with cervical cancer?
9. What questions or concerns do you have about cervical cancer and its management?

   R: I wish the government could reduce the cost for the service especially for the poor ones, as the diseases is affecting a lot of women. The government should find vaccine to protect other women.

Appendix ii (a): Interview Guide - English Version

"For non-adherent cervical cancer patients attending Bugando Oncology Clinic"

Identification number – BMC Onco 11 Date for an in-depth interview – 02/09/2023

A. Participant's Socio-demographic Information

1. Age (years) – 53
2. Highest education level reached – No formal education
3. Occupation – Peasant
4. Religious denomination – Pentecost
5. Marital status – Married
6. Residence (district, region) – Ilemela, Mwanza

B. Exploring of healthcare-seeking behaviors

1. Nowadays there are many health problems affecting people of the lake zone, and cancer is on the increase, mention types of cancers?

   R: Cervical cancer

Probe: (If cervical cancer is not mentioned) How about cervical cancer?

2. What are the symptoms of cervical cancer?

   R: Bleeding, vaginal discharge, back pain, diarrhea, loss of appetite

Probe: What symptoms or concerns led you to seek healthcare for your cervical cancer?

3. Tell me any instances where you tried to treat yourself for cervical cancer or its symptoms.

Probe: What led you to believe that self-treatment was necessary or preferable?

4. What other alternative or complementary treatments have you sought?

   R: Attending to traditional healers

Probe: Please, explain your experiences with these treatments.

Probe: How did you make a decision about which treatment to pursue?

5. What barriers or challenges have you faced in seeking healthcare for your cervical cancer?

   R: Attending nearby hospital but no relief

Probe: How have you managed to overcome those challenges?

6. Describe sociocultural factors that have influenced your decision-making for cancer care.

Probe: What specific misconceptions you have heard about cervical cancer treatment?

7. What advice would you give to other women who may be experiencing symptoms or concerns related to cervical cancer?
8. Is there anything else you would like to share about your experience with seeking healthcare for your cervical cancer?

C. Identifying factors influencing non-adherence

1. What were your initial thoughts and feelings when you were diagnosed with cervical cancer?

   R: It pained me a lot

2. What are the treatment options for cervical cancer?

Probe: Which treatment did your healthcare provider recommend for you?

3. What specific conditions you were given to follow during your treatment for cervical cancer?

   R: No conditions

Probe: Were they clear and easy to understand?

4. What did your healthcare provider explain to you regarding your treatment regimen?

   R: I don’t know

Probe: How long would it take you to complete your planned treatments?

Probe: How frequently were you supposed to receive your treatments?

5. What challenges did you face in adhering to your treatment for cervical cancer?

Probe: How have you managed these challenges?

6. What is your understanding of the importance of adherence to treatment plans for cervical cancer?
7. During your attendance for cancer care in Bugando Oncology Clinic, have you ever received teachings or materials (pictures, flyers etc.) to help improve your adherence?

   R: Not yet
8. Is there anything else you would like to share about your experience with cervical cancer?
9. What questions or concerns do you have about cervical cancer and its management?

Appendix ii (a): Interview Guide - English Version

"For non-adherent cervical cancer patients attending Bugando Oncology Clinic"

Identification number – BMC Onco 12 Date for an in-depth interview – 03/09/2023

A. Participant's Socio-demographic Information

1. Age (years) – 50
2. Highest education level reached – No formal education
3. Occupation – Peasant
4. Religious denomination – No religion
5. Marital status – Married
6. Residence (district, region) – Kwimba, Mwanza

B. Exploring of healthcare-seeking behaviors

1. Nowadays there are many health problems affecting people of the lake zone, and cancer is on the increase, mention types of cancers?

   R: Cervical cancer

Probe: (If cervical cancer is not mentioned) How about cervical cancer?

2. What are the symptoms of cervical cancer?

   R: Bleeding, fistula, anemia

Probe: What symptoms or concerns led you to seek healthcare for your cervical cancer?

   R: Bleeding and fistula

3. Tell me any instances where you tried to treat yourself for cervical cancer or its symptoms.

Probe: What led you to believe that self-treatment was necessary or preferable?

4. What other alternative or complementary treatments have you sought?

R: Traditional medicine to easy the symptoms

Probe: Please, explain your experiences with these treatments.

Probe: How did you make a decision about which treatment to pursue?

5. What barriers or challenges have you faced in seeking healthcare for your cervical cancer?

Probe: How have you managed to overcome those challenges?

6. Describe sociocultural factors that have influenced your decision-making for cancer care.

   R: as the traditional medicine didn't work that's we went to hospital

Probe: What specific misconceptions you have heard about cervical cancer treatment?

7. What advice would you give to other women who may be experiencing symptoms or concerns related to cervical cancer?

   R: to go to hospital

8. Is there anything else you would like to share about your experience with seeking healthcare for your cervical cancer?

C. Identifying factors influencing non-adherence

1. What were your initial thoughts and feelings when you were diagnosed with cervical cancer?

   R: I felt afraid

2. What are the treatment options for cervical cancer?

   R: Radiotherapy

Probe: Which treatment did your healthcare provider recommend for you?

3. What specific conditions you were given to follow during your treatment for cervical cancer?

   R: To eat fruit and vegetables

Probe: Were they clear and easy to understand?

4. What did your healthcare provider explain to you regarding your treatment regimen?

R: To be treated for 25 fractions, and consult a doctor on Thursdays

Probe: How long would it take you to complete your planned treatments?

Probe: How frequently were you supposed to receive your treatments?

5. What challenges did you face in adhering to your treatment for cervical cancer?
   R: I was anemic, therefore stopped radiotherapy for one week
   R: Diarrhea, burning sensation as the side effects of chemotherapy

Probe: How have you managed these challenges?

6. What is your understanding of the importance of adherence to treatment plans for cervical cancer?

   R: I feel some relief although I still have diarrhea and fistula

7. During your attendance for cancer care in Bugando Oncology Clinic, have you ever received teachings or materials (pictures, flyers etc.) to help improve your adherence?

   R: I attended once in the teachings

8. Is there anything else you would like to share about your experience with cervical cancer?

9. What questions or concerns do you have about cervical cancer and its management?

   R: When will my fistula stop?

Appendix ii (a): Interview Guide - English Version

"For non-adherent cervical cancer patients attending Bugando Oncology Clinic"

Identification number – BMC Onco 13 Date for an in-depth interview – 29/08/2023

A. Participant's Socio-demographic Information

1. Age (years) – 44
2. Highest education level reached – Standard four
3. Occupation – Peasant
4. Religious denomination – Pentecost
5. Marital status – Married
6. Residence (district, region) – Tarime, Mara

B. Exploring of healthcare-seeking behaviors

1. Nowadays there are many health problems affecting people of the lake zone, and cancer is on the increase, mention types of cancers?

   R: Cervical cancer

Probe: (If cervical cancer is not mentioned) How about cervical cancer?

2. What are the symptoms of cervical cancer?

   R: Heavy bleeding

Probe: What symptoms or concerns led you to seek healthcare for your cervical cancer?

3. Tell me any instances where you tried to treat yourself for cervical cancer or its symptoms.

Probe: What led you to believe that self-treatment was necessary or preferable?

4. What other alternative or complementary treatments have you sought?

Probe: Please, explain your experiences with these treatments.

Probe: How did you make a decision about which treatment to pursue?

5. What barriers or challenges have you faced in seeking healthcare for your cervical cancer?

   R: Financial barriers

Probe: How have you managed to overcome those challenges?

6. Describe sociocultural factors that have influenced your decision-making for cancer care.

Probe: What specific misconceptions you have heard about cervical cancer treatment?

R: I heard that Treatment involve burning with fire

7. What advice would you give to other women who may be experiencing symptoms or concerns related to cervical cancer?

   R: I will tell her to go to the hospital

8. Is there anything else you would like to share about your experience with seeking healthcare for your cervical cancer?

C. Identifying factors influencing non-adherence

1. What were your initial thoughts and feelings when you were diagnosed with cervical cancer?

   R: I was afraid but after they told me that it is treatable, I felt fine

2. What are the treatment options for cervical cancer?

Probe: Which treatment did your healthcare provider recommend for you?

R: Radiotherapy for 25 days, 3 chemotherapy and brachytherapy

3. What specific conditions you were given to follow during your treatment for cervical cancer?

   R: No conditions

Probe: Were they clear and easy to understand?

4. What did your healthcare provider explain to you regarding your treatment regimen?

Probe: How long would it take you to complete your planned treatments?

Probe: How frequently were you supposed to receive your treatments?

5. What challenges did you face in adhering to your treatment for cervical cancer?
   R: No challenges

Probe: How have you managed these challenges?

6. What is your understanding of the importance of adherence to treatment plans for cervical cancer?

   R: I feel good with my health, I sleep well

7. During your attendance for cancer care in Bugando Oncology Clinic, have you ever received teachings or materials (pictures, flyers etc.) to help improve your adherence?

   R: I have attended some teachings

8. Is there anything else you would like to share about your experience with cervical cancer?

   R: Just wishing you a good heart to serve cancer patients

9. What questions or concerns do you have about cervical cancer and its management?

Appendix ii (a): Interview Guide - English Version

"For non-adherent cervical cancer patients attending Bugando Oncology Clinic"

Identification number – BMC Onco 14 Date for an in-depth interview – 04/09/2023

A. Participant's Socio-demographic Information

1. Age (years) – 39
2. Highest education level reached – Standard seven
3. Occupation – Peasant
4. Religious denomination – Roma Catholic
5. Marital status – Not Married/Single
6. Residence (district, region) – Kahama, Shinyanga

B. Exploring of healthcare-seeking behaviors

1. Nowadays there are many health problems affecting people of the lake zone, and cancer is on the increase, mention types of cancers?

   R: Cervical cancer, Breast cancer

Probe: (If cervical cancer is not mentioned) How about cervical cancer?

2. What are the symptoms of cervical cancer?

   R: Post coital bleeding, difficult in urination and defecation

Probe: What symptoms or concerns led you to seek healthcare for your cervical cancer?

3. Tell me any instances where you tried to treat yourself for cervical cancer or its symptoms.

Probe: What led you to believe that self-treatment was necessary or preferable?

4. What other alternative or complementary treatments have you sought?

Probe: Please, explain your experiences with these treatments.

Probe: How did you make a decision about which treatment to pursue?

5. What barriers or challenges have you faced in seeking healthcare for your cervical cancer?

   R: I went to hospital and they said I was having UTI, and they kept treating me with antibiotics

   R: Financial barriers as I didn't have enough money to cover hospital expenses

Probe: How have you managed to overcome those challenges?

6. Describe sociocultural factors that have influenced your decision-making for cancer care.

   R: My family supported me

Probe: What specific misconceptions you have heard about cervical cancer treatment?

7. What advice would you give to other women who may be experiencing symptoms or concerns related to cervical cancer?
8. R: To go early to the hospital
9. Is there anything else you would like to share about your experience with seeking healthcare for your cervical cancer?

C. Identifying factors influencing non-adherence

1. What were your initial thoughts and feelings when you were diagnosed with cervical cancer?

   R: I was much stressed

2. What are the treatment options for cervical cancer?

   R: Surgery and Radiotherapy

Probe: Which treatment did your healthcare provider recommend for you?

R: Radiotherapy

3. What specific conditions you were given to follow during your treatment for cervical cancer?

   R: Frequently eating so as to maintain my Hb level

Probe: Were they clear and easy to understand?

4. What did your healthcare provider explain to you regarding your treatment regimen?

   R: External Radiotherapy for 25 days followed by brachytherapy for 3 times in three weeks

Probe: How long would it take you to complete your planned treatments?

Probe: How frequently were you supposed to receive your treatments?

5. What challenges did you face in adhering to your treatment for cervical cancer?
   R: No challenge

Probe: How have you managed these challenges?

6. What is your understanding of the importance of adherence to treatment plans for cervical cancer?

   R: I feel somehow better

7. During your attendance for cancer care in Bugando Oncology Clinic, have you ever received teachings or materials (pictures, flyers etc.) to help improve your adherence?

   R: Yes I always attend morning teachings

8. Is there anything else you would like to share about your experience with cervical cancer?

9. What questions or concerns do you have about cervical cancer and its management?

   R: I don’t have any question as we are always get the teachings

Appendix ii (a): Interview Guide - English Version

"For non-adherent cervical cancer patients attending Bugando Oncology Clinic"

Identification number – BMC Onco 15 Date for an in-depth interview – 02/09/2023

A. Participant's Socio-demographic Information

1. Age (years) – 50
2. Highest education level reached – Standard seven
3. Occupation – Peasant
4. Religious denomination – Pentecost
5. Marital status – Divorced
6. Residence (district, region) – Bwiru, Ilemela, Mwanza.

B. Exploring of healthcare-seeking behaviors

1. Nowadays there are many health problems affecting people of the lake zone, and cancer is on the increase, mention types of cancers?

   R: Cervical cancer, throat cancer, eye cancer, bladder cancer, breast cancer

Probe: (If cervical cancer is not mentioned) How about cervical cancer?

2. What are the symptoms of cervical cancer?

   R: Non-stop bleeding,

Probe: What symptoms or concerns led you to seek healthcare for your cervical cancer?

R: Non-stop bleeding

3. Tell me any instances where you tried to treat yourself for cervical cancer or its symptoms.

   R: I didn't try any, I went to hospital

Probe: What led you to believe that self-treatment was necessary or preferable?

4. What other alternative or complementary treatments have you sought?

   R: No

Probe: Please, explain your experiences with these treatments.

Probe: How did you make a decision about which treatment to pursue?

5. What barriers or challenges have you faced in seeking healthcare for your cervical cancer?

   R: No it was just me who delayed to seek treatment

Probe: How have you managed to overcome those challenges?

6. Describe sociocultural factors that have influenced your decision-making for cancer care.

   R: My dad died from cancer, so I had some experience about cancer that made me go for treatment after noticed the symptoms

Probe: What specific misconceptions you have heard about cervical cancer treatment?

7. What advice would you give to other women who may be experiencing symptoms or concerns related to cervical cancer?

   R: I have started to tell three women with cervical cancer to go to the hospital

8. Is there anything else you would like to share about your experience with seeking healthcare for your cervical cancer?

   R: I had challenges with financial barrier especially to buy drugs and seen patients from outside Mwanza suffer to find a place to stay

C. Identifying factors influencing non-adherence

1. What were your initial thoughts and feelings when you were diagnosed with cervical cancer?

   R: I felt pain, but I presented myself before God

2. What are the treatment options for cervical cancer?

   R: Chemotherapy and Radiotherapy

Probe: Which treatment did your healthcare provider recommend for you?

R: Chemotherapy and radiotherapy

3. What specific conditions you were given to follow during your treatment for cervical cancer?

   R: To stay away from fire, not to do heavy work, to eat on time

Probe: Were they clear and easy to understand?

4. What did your healthcare provider explain to you regarding your treatment regimen?

   R: I was told to come for 25 days for treatment

Probe: How long would it take you to complete your planned treatments?

Probe: How frequently were you supposed to receive your treatments?

5. What challenges did you face in adhering to your treatment for cervical cancer?
   R: Machine breakdown for two days
   R: Financial challenge
   R: I suffered anemia

Probe: How have you managed these challenges?

6. What is your understanding of the importance of adherence to treatment plans for cervical cancer?

   R: There is benefit because I see some positive changes

7. During your attendance for cancer care in Bugando Oncology Clinic, have you ever received teachings or materials (pictures, flyers etc.) to help improve your adherence?

   R: I once attended the teaching

8. Is there anything else you would like to share about your experience with cervical cancer?

9. What questions or concerns do you have about cervical cancer and its management?

   R: What next about the management of my disease?
