# Supplementary material for: Healthcare-seeking behaviors and factors influencing non-adherence among cervical cancer patients attending Bugando Oncology Clinic in Mwanza, Tanzania: A qualitative Phenomenological study
Source: PLoS One. 2025 Mar 26;20(3):e0317609. doi: 10.1371/journal.pone.0317609 (PMC11940420; doi:10.1371/journal.pone.0317609)
Supplement: S3 File — (DOCX) [file pone.0317609.s003.docx]

**EXPLORING HEALTHCARE-SEEKING BEHAVIORS AND FACTORS INFLUENCING NON-ADHERENCE AMONG CERVICAL CANCER PATIENTS ATTENDING BUGANDO ONCOLOGY CLINIC IN MWANZA, TANZANIA.**

**A code book for in-depth interviews.**

| **S/N** | **THEMES** | **THEMES DESCRIPTION** |
| --- | --- | --- |
| **1** | **A: Demographic Characteristics of The Participants**   - Age - Gender - Religion/denomination - Highest Education level reached - Marital status - Residence - Occupation | Demographic information on each participant |
| **2**  3 | **B: Exploring of healthcare-seeking behaviors**   - Types of cancer that informants know - (If cervical cancer is not mentioned) How about cervical cancer? - Informants understanding about Symptoms of cervical cancer - Symptoms or concerns that led them to seek healthcare for your cervical cancer - Any instances where they tried to treat themselves for cervical cancer or its symptoms - Reasons why they believed that self-treatment was necessary or preferable? - Other treatments or complementary treatments carried out - Informant’s experiences with these kinds of treatments - Decision making about which treatment to pursue? - Barriers or challenges faced in seeking healthcare for cervical cancer - How they managed to overcome those challenges - Informants’ description of the sociocultural factors that have influenced their decision making for cancer care - Specific misconceptions they have heard about cervical cancer treatment - Informant’s advices to other women who may be experiencing symptoms or concerns related to cervical cancer - Anything else that informants would like to share about their experience with seeking healthcare for cervical cancer | To pursue awareness and understanding of cervical cancer and its treatment among clients |
|  | **C**: **Identifying factors influencing non-adherence**   - Informant’s initial thoughts and feelings when diagnosed with cervical cancer - Treatment options for cervical cancer - Treatment that health care providers recommended for them - Specific conditions that informants were instructed to follow during treatment for cervical cancer - Clear and easy to understand? - Things that healthcare provider explain to them regarding treatment regimen - How long would it take a client to complete planned treatments - How frequently a client was supposed to receive treatments - Challenges they faced in adhering to treatments of cervical cancer - How these challenges were managed - Informant’s understandings of the importance for adhering to treatment plans for cervical cancer - Any materials or teachings received during their attendance for cancer care in Bugando Oncology Clinic. (Pictures, flyers etc.) in order to help improve their adherence - Anything else that informants would like to share about their experience with cervical cancer - Questions or concerns about cervical cancer and its management | To understand measures, perceptions and client’s readiness to adhere to cervical cancer treatment, observe challenges and obstacles they face after being diagnosed. |

- Stands for Parent Node
- Stands for Child Node
