# Supplementary material for: Healthcare-seeking behaviors and factors influencing non-adherence among cervical cancer patients attending Bugando Oncology Clinic in Mwanza, Tanzania: A qualitative Phenomenological study
Source: PLoS One. 2025 Mar 26;20(3):e0317609. doi: 10.1371/journal.pone.0317609 (PMC11940420; doi:10.1371/journal.pone.0317609)
Supplement: Attachment — Submitted filename: Comment Reviewer Healthcare-seeking behaviors.pdf [file pone.0317609.s005.pdf]

Healthcare-seeking behaviors and factors influencing non-adherence among cervical cancer patients attending Bugando oncology clinic in Mwanza, Tanzania: Qualitative phenomenological study.

Comments to the Author

Congratulations on the submitted manuscript. The topic is timely and will be of interest to the readers of the journal. However, a few changes are suggested to improve the clarity of this manuscript. I have several recommendations and questions about the manuscript.

No	Page, Para	Descriptions of item	Comment
1.		**Abstract**<br>Findings:<br>The findings revealed delayed healthcare-seeking behavior and poor adherence to most of the study participants seeking medical care at nearby health facilities with limited capacity for correct cervical cancer diagnosis. Some turned to self medications including traditional remedies.	Need to paraphrase because the similarity index is 29 using Turnitin<br><br>Please harmonize the statements
2	2,59	**Introduction**<br>Among the estimated 40,464 individuals diagnosed with cancer in 2020, cervical cancer constituted 25.3% of all newly identified cases.	Please add a reference to support this statement.
3.	5,120<br><br>8,179	**Methodology**<br><br>In this research we took into consideration the process known as 'bracketing'<br><br>Kiswahili transcripts were then translated into English for further processing.	Explain the types of Bracketing used here, Descriptive Bracketing or Reflexive Bracketing?<br><br>Explain in detail the further processing after translation, the English transcripts regarding thematic analysis, coding and report writing in subheading, to make clearer.

4.	9,206	**Results**<br><br>These individuals included women aged 35–39 (n=2), 40–49 (n=3), 50–59 (n=8), and 60–65 (n=2). Their education levels ranged from no formal education (n=5) to primary school dropout (n=3), primary school completion (n=6), and secondary school dropout (n=1). Marital statuses included single (n=1), married (n=6), separated (n=1), divorced (n=3), and widowed (n=4). Religious affiliations varied, with informants identifying as having no religion (n=2), Catholic (n=6), Mennonite (n=1), Pentecostal (n=3), Baptist (n=1), and Muslim (n=2).	 <br>Please write in percentages also<br>n(%)
5.		**Discussion**	Highlight the implication of the results to health policy<br><br>How do the findings add to the body of scientific knowledge on the issue?
6		**Conclusion**	The conclusion very general.<br>The author should specify what was the 'valuable information' in the conclusion and briefly describe the importance of the 'valuable information''.
7.		**References**	16/ 34 of the references 5 year above.<br>Recommended for 5 years and under
