# Supplementary material for: Healthcare-seeking behaviors and factors influencing non-adherence among cervical cancer patients attending Bugando Oncology Clinic in Mwanza, Tanzania: A qualitative Phenomenological study
Source: PLoS One. 2025 Mar 26;20(3):e0317609. doi: 10.1371/journal.pone.0317609 (PMC11940420; doi:10.1371/journal.pone.0317609)
Supplement: Attachment — Submitted filename: Response to Reviewers.docx [file pone.0317609.s007.docx]

**RESPONSE TO REVIEWERS’ COMMENTS**

| **No.** | **Page**  **para** | **Description of item** | **Comments** | **Response** |
| --- | --- | --- | --- | --- |
| 1. |  | **Abstract** Findings: The findings revealed delayed healthcare-seeking behaviours and poor adherence to most of the study participants seeking medical care at nearby health facilities with limited capacity for correct cervical cancer diagnosis. Some turned to self-medication including traditional remedies | Need to paraphrase because the similarity index is 29 using Turnitin Please harmonize the statements | The statement has been harmonized as follows: The study revealed poor adherence to treatment-seeking by most of the study participants in the form of delayed health-seeking at nearby health facilities. The limited capacity for correct cervical cancer diagnosis also pushed some of them to turn to self-medication including traditional remedies. |
| 2. | 2,59 | **Introduction** Among the estimated 40,464 individuals diagnosed with cancer in 2020, cervical cancer constituted 25.3% of all newly identified cases. | Please add a reference to support this statement. | The reference is provided as suggested. |
| 3 | 5,120  8,179 | **Methodology** In this research we took into consideration the process known as ‘bracketing’ Kiswahili transcripts were then translated into English for further processing. | Explain the types of Bracketing used here, Descriptive Bracketing or Reflexive Bracketing? Explain in detail the further processing after translation, the English transcripts regarding thematic analysis, coding and report writing in subheading, to make clearer. | Both types of bracketing (i.e., descriptive and reflexive) were used in this study.  Descriptive bracketing involved suspending or setting aside our pre-conceived notions, biases and assumptions about the world to better understand the phenomenon being studied. This process allowed us to approach our informants with an open mind and focus on how experiences appear in themselves, without being influenced by external judgements or interpretations.  The goal of descriptive bracketing was to capture the essence of informants’ experience as purely as possible. By doing so, we were able to gain deeper insights into the lived experiences of individuals and understand the meaning they attach to those experiences.  Reflexive bracketing involved acknowledging that complete objectivity is impossible. Instead, we tried to eliminate biases, by actively reflecting on our own influence on the research process. This involved being aware of how our backgrounds[, experiences, and assumptions might shape our interpretations and interactions with the data.](https://journals.sagepub.com/doi/pdf/10.1177/16094069231191635) Reflexive bracketing was about transparency and reflexivity, making the research process more rigorous by explicitly acknowledging and accounting for the researcher’s role.  Both types of bracketing were essential in this phenomenological research to ensure that the findings are as true to the participants’ experiences as possible, while also maintaining the integrity and credibility of the research process.  Data processing and analysis has now been explained separately and in detail under subheading to make it clearer. |
| 4. | 9,206 | **Results** | These individuals included women aged 35–39 (n=2), 40–49 (n=3), 50–59 (n=8), and 60–65 (n=2). Their education levels ranged from no formal education (n=5) to primary school dropout (n=3), primary school completion (n=6), and secondary school dropout (n=1). Marital statuses included single (n=1), married (n=6), separated (n=1), divorced (n=3), and widowed (n=4). Religious affiliations varied, with informants identifying as having no religion (n=2), Catholic (n=6), Mennonite (n=1), Pentecostal (n=3), Baptist (n=1), and Muslim (n=2). Please write in percentages also n (%) | These individuals included women aged 35–39 (n=2, 13.3%), 40–49 (n=3, 20.0%), 50–59 (n=8,53.3%), and 60–65 (n=2, 13.3%). Their education levels ranged from no formal education (n=5, 33.3%) to primary school dropout (n=3, 20.0%), primary school completion (n=6, 40.0%), and secondary school dropout (n=1, 6.7%). Marital statuses included single (n=1, 6.7%), married (n=6, 40.0%), separated (n=1, 6.7%), divorced (n=3, 20.0%), and widowed (n=4, 26.7%). Religious affiliations varied, with informants identifying as having no religion (n=2, 13.3%), Catholic (n=6, 40.0%), Mennonite (n=1, 6.7%), Pentecostal (n=3, 20.0%), Baptist (n=1, 6.7%), and Muslim (n=2, 13.3%). |
| 5 | 5. | **Discussion** | Highlight the implication of the results to health policy How do the findings add to the body of scientific knowledge on the issue? | The study revealed key barriers to cervical cancer care in Tanzania, including inadequate healthcare infrastructure, financial constraints, equipment malfunctions, and poor patient education. Health policy should focus on improving diagnostic capacity, ensuring financial support, maintaining treatment equipment, and enhancing patient education to improve treatment adherence and outcomes.  The findings expand the body of scientific knowledge by identifying specific, real-world barriers to cervical cancer care in Tanzania, such as limited diagnostic capacity, financial constraints, and equipment malfunctions. These insights deepen the understanding of how systemic factors contribute to treatment non-adherence. Additionally, the study highlights the crucial role of patient education in improving adherence, providing evidence that can inform targeted interventions. By focusing on the unique challenges in a low-resource setting, the research enriches global discussions on cancer care disparities and offers actionable data for improving healthcare policy and practices in similar contexts. |
| 6. | 6. | **Conclusion** | The conclusion very general. The author should specify what was the 'valuable information' in the conclusion and briefly describe the importance of the 'valuable information". | The study's ‘valuable information’ lies in identifying the key factors contributing to non-adherence among cervical cancer patients: inadequate healthcare infrastructure, financial constraints, malfunctioning radiotherapy equipment, and insufficient patient education. This information is valuable as it highlights specific barriers that prevent effective cancer care and lead patients to seek self-medication including traditional remedies. The importance of addressing these barriers cannot be overstated—improving healthcare access, funding, equipment reliability, and patient education are essential steps to enhance treatment adherence, reduce the burden on cancer services, and improve patient outcomes. These insights provide actionable targets for health policy interventions aimed at improving cervical cancer care in resource-limited settings. |
| 7 | 7. | **References** | 16/ 34 of the references 5 year above.  Recommended for 5 years and under | The said references have been reworked as recommended. |
